# Robotic telepresence in acute care facilities in community hospitals in Mexico

**DOI:** 10.1186/cc9903

**Published:** 2011-03-11

**Authors:** G Vazquez de Anda, S Larraza Rico, L De la Cruz Avila, S Resendiz, M Escudero, R Tapia Rodriguez, Y Gutierrez Mariano, G Diaz Diego, J Mejia Nava, R Camacho Beiza

**Affiliations:** 1 Universidad Autonoma del Estado de Mexico, Toluca, Mexico; 2 Hospital Materno Perinatal Monica Pretelini del Instituto de Salud del Estado de Mexico, Toluca, Mexico

## Introduction

Telepresence using robots (TPR) in acute care facilities (ACF) is being increasingly accepted as a new and practical way of solving the shortage of intensivists in community hospitals (CH) where there are not specialists available 24/7 [1,2]. The main objective of this study is to show the early experience of using TPR in four CH in Mexico.

## Methods

Four CH with 60 beds each were fully equipped with a telepresence system that includes: high-speed connectivity, wireless Internet access, a RP-7i robot (INTOUCH HEALTH, Santa Barbara, CA, USA), three computers (laptops) per hospital and one central computer based at the MPH. Additionally, all four CH has a specific team for technical support. The CH cover medical care to people without social security and are far away from the capital city at not less than 42 km (Tenancingo 42 km, Atlacomulco 63 km, Valle de Bravo 85 km and Tejupilco 90 km). The program includes a team of certified intensivists that is based at MPH. The task of the team is to assist physicians in ACF, which includes the emergency room (ER), ICUs and the operating room (OR). The program includes: rounds at ACF every day three times a day 24/7 and every time that it is needed. The MPH team gives assistance in: advanced trauma life support (ATLS), advanced cardiovascular life support (ACLS), advance life support for pregnant women (APLS), neurological support (NS), rapid response team support (RRTS), and air transportation assistance (AT). Hospitalization, discharge, transfer to high-care hospitals, and mortality were recorded.

## Results

From 1 May to 30 November 2010, 319 patients were attended by the MPH team: 54 ER patients, 16 ATLS and 66 ACLS interventions, 106 ICU patients, 17 interventions as RRTS, 76 APLS, six AT, 27 OR assistances, and 22 NS. Forty-five percent of patients were discharged home, 25% were transferred to high-care hospitals, 9% were still in hospital and the total mortality rate was 19%.

## Conclusions

This preliminary report shows that the practice of telepresence using robots in ACF is feasible at community hospitals in Mexico. Additionally, we observed minimal resistance to the expertise given by the MPH team.
